# Linking Neuronal Ensembles by Associative Synaptic Plasticity

**DOI:** 10.1371/journal.pone.0020486

**Published:** 2011-06-29

**Authors:** Qi Yuan, Jeffry S. Isaacson, Massimo Scanziani

**Affiliations:** 1 Department of Neurobiology, Center for Neural Circuits and Behavior, University of California at San Diego, La Jolla, California, United States of America; 2 Department of Neuroscience, Center for Neural Circuits and Behavior, University of California at San Diego, La Jolla, California, United States of America; 3 Howard Hughes Medical Institute, Center for Neural Circuits and Behavior, University of California at San Diego, La Jolla, California United States of America; The Research Center of Neurobiology-Neurophysiology of Marseille, France

## Abstract

Synchronized activity in ensembles of neurons recruited by excitatory afferents is thought to contribute to the coding information in the brain. However, the mechanisms by which neuronal ensembles are generated and modified are not known. Here we show that in rat hippocampal slices associative synaptic plasticity enables ensembles of neurons to change by incorporating neurons belonging to different ensembles. Associative synaptic plasticity redistributes the composition of different ensembles recruited by distinct inputs such as to specifically increase the similarity between the ensembles. These results show that in the hippocampus, the ensemble of neurons recruited by a given afferent projection is fluid and can be rapidly and persistently modified to specifically include neurons from different ensembles. This linking of ensembles may contribute to the formation of associative memories.

## Introduction

In the brain features about the external world are represented by the activity of ensembles of neurons rather than by individual neurons [1], [2], [3]. In the hippocampus, for example, the firing of ensembles of “place” cells represents information about specific spatial locations in a manner that is more accurate than the representation provided by any individual cell within the ensemble [4], [5], [6]. The composition of an ensemble within a neuronal population is determined by the specific activity pattern of afferent inputs to that population and by the strength of the synapses between afferents and their target population. Experience-dependent changes in synaptic strength are thus likely to strongly modify the composition of neuronal ensembles, and hence critically affect the representation of a given afferent input. Thus, associative forms of synaptic plasticity resulting from the co-activation of distinct afferent inputs [7] may lead to the generation of novel ensembles whose composition is a combination of the two associated ensembles. While the rules governing changes in synaptic strength at the cellular level are well established [8], how large ensembles of neurons are transformed by associative synaptic plasticity is unclear. In this study we address how associative plasticity modifies the composition of neuronal ensembles recruited by independent afferent pathways.

## Results

**Figure 1:**
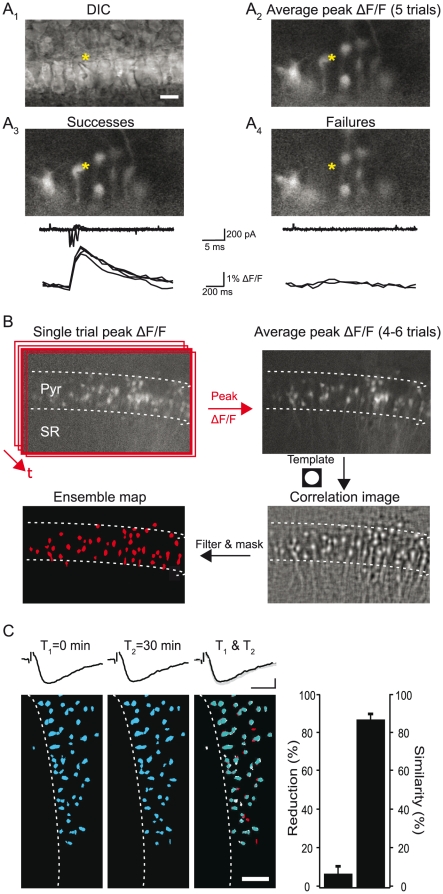
Imaging CA1 pyramidal cell ensembles recruited by stimulation of Schaffer collateral afferent inputs. A, Calcium transients in Oregon Green-1 loaded CA1 pyramidal cells are action-potential dependent. A_1_, DIC image of the pyramidal cell layer. The pyramidal cell marked by a yellow asterisk was recorded in the loose patch configuration and SC inputs were evoked via a stimulating electrode in stratum radiatum. Stimulus strength was set at threshold for evoking spikes in the targeted cell. Scale bar, 20 µm. A_2_, SC stimulation evokes calcium transients revealed by the ΔF/F image averaged across 6 stimulus trials. A_3,_ Average dF/F image of 4 trials in which a calcium transient was detected in the targeted cell (Successes). Traces of individual trials show loose patch recordings of action potentials from the targeted cell (top) and time course of the dF/F signal of the same cell. A_4_, average dF/F image of 2 trials in which a calcium transient was not evoked (Failures). Traces indicate that the failure to evoke action potentials on single trials (top) did not generate calcium transients in the targeted cell. Calcium transients were always associated with spiking in all cells tested with loose patch recording (n = 6). B, Steps diagramming methods used to construct activity maps of cell ensembles. C, Activity maps of SC-evoked cell ensembles are stable over time. Left, Representative experiment illustrating cell ensembles recruited by SC stimulation at two time points (T1 and T2, 30 minute interval). Activated neurons in the pyramidal cell layer are color-coded blue and field EPSPs recorded in stratum radiatum during each imaging period are shown above. The activity maps and field EPSPs from the two periods are overlaid (T1 + T2, image color code: blue cells are recruited during both imaging periods, white cells are those recruited during T1 but absent during T2, red cells are those recruited during T2 but absent during T1). Scale bar for activity maps, 50 µm. Right, summary (n = 5) of the stability of cell ensembles over a 30 min time period.

We visualized ensembles of CA1 pyramidal cells by imaging hippocampal slices bulk loaded with the calcium indicator dye Oregon Green-1 AM [9], [10]. Action potentials in dye-loaded cells generated somatic calcium transients (Fig. 1A) that we used to identify cells recruited by Schaffer collateral (SC) stimulation (the SC ensemble). We created activity maps of recruited cells by averaging the peak dF/F images collected from several consecutive stimulus trials (Figure 1B). Activity maps of cell ensembles were stable over time (Fig. 1C). Over a 30 min period, although the number of cells recruited showed a small reduction (6.7±4.2%; n = 5) the identity of the remaining cells was largely unchanged (86.6±2.9% similarity).

**Figure 2:**
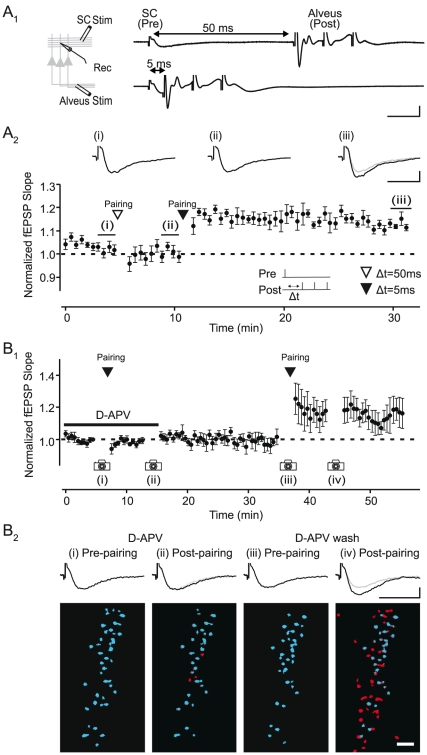
Timing-dependent associative synaptic plasticity enlarges active neural ensembles. A_1_ Left, recording configuration. Right, induction protocol for studying timing-dependent plasticity. Example traces show the SC-evoked field EPSP followed 50 ms (top) or 5 ms (bottom) later by alveus stimulation (3 pulses, 100 Hz). A_2_, Summary plot of timing-dependent LTP of SC fEPSPs induced by paired pre- and postsynaptic activity (n = 5). Single SC-evoked EPSPs (pre) were paired with brief trains of alveus stimulation (post, 3 pulses, 100 Hz) for 30 trials at 0.5 Hz. Pairing of alveus stimuli 50 ms following presynaptic activity (open triangle) had no effect on the fEPSP, while subsequent pairing using a 5 ms delay led to stable LTP. Top, representative fEPSPs recorded at the time points indicated on the summary plot. B, Pairing-induced LTP is NMDAR-dependent and enhances the number of pyramidal cells belonging to the SC ensemble. B_1_, Pairing SC and alveus stimulation (5 ms delay) in the presence of D-APV (50 µM) has no effect on the fEPSP, while subsequent pairing following drug washout elicits LTP (n = 5). B_2_, Pairing-induced LTP of fEPSPs is accompanied by an enlargement of the SC ensemble. Activity maps of SC-evoked CA1 cell ensembles from a representative experiment. Images and corresponding fEPSPs were acquired during the periods indicated by cameras in B_1_. Activated neurons in the pyramidal cell layer are color-coded blue and cells added after pairing are colored red. Scale bar for activity maps, 50 µm. Scale bars for fEPSPs, 0.5 mV and 20 ms.

We first asked whether a simple and direct form of associative synaptic plasticity could enlarge SC ensembles to selectively incorporate neurons of a defined population. To address this question, we designed a protocol to induce synaptic plasticity by pairing presynaptic activity with postsynaptic depolarization of a specific population of CA1 pyramidal cells. We recorded the field EPSP in stratum radiatum evoked by SC stimulation and placed a stimulating electrode in the alveus (Fig. 2A_1_), the dense fiber tract mainly composed of CA1 pyramidal cell axons. We thus used alveus stimulation to provide postsynaptic depolarization via antidromic activation of CA1 pyramidal cells. In this configuration, brief bursts of alveus stimulation (3 pulses, 100 Hz) delivered 5 ms after activation of SCs led to a stable, long-term potentiation (LTP) of the field EPSP (114.1±2.5%; n = 5; p = 0.005; Fig. 2A_2_). This synaptic plasticity was timing-dependent [11], since delivering the alveus stimulus 50 ms following presynaptic (SC) stimulation failed to increase the field EPSP (95.7±1.6%; n = 5; p = 0.060; Fig. 2A_2_). The associative, timing-dependent LTP of the field EPSP was NMDA receptor-dependent since it was blocked by the specific antagonist D-APV (50 µM; 99.2±1.0%; n = 4; p = 0.475; Fig. 2B_1_). We combined these recordings of synaptic transmission with imaging of CA1 cells and found that pairing of alveus and SC stimulation led to an NMDAR-dependent increase in the number of cells in the SC ensemble (32.9±9.8%; n = 6; p<0.001; Fig. 2B_2_).

**Figure 3:**
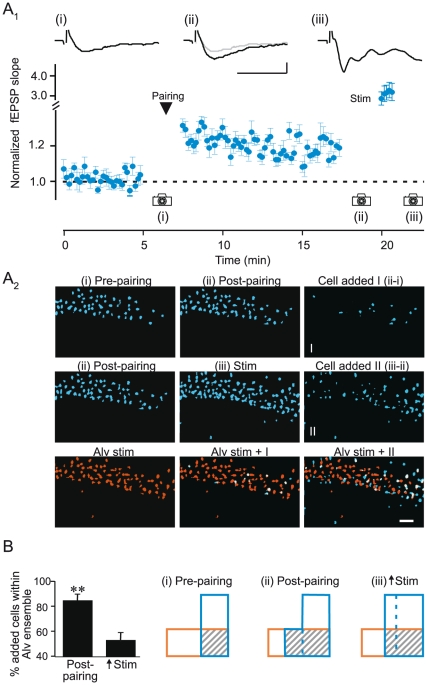
Pairing-induced synaptic plasticity selectively recruits cells from a defined population. A_1_, Summary plot showing increases in fEPSPs following pairing-induced LTP and subsequent increase in SC stimulus strength (n = 6). Example fEPSPs (top traces) from one experiment at the indicated time points (scale bars, 0.2 mV, 20 ms). A_2_, Cell activity maps from one experiment at the indicated time points (cameras, scale = 50 µm). Top row, Images show cells activated by the SC stimulation (blue) before (i) and after (i) pairing along with the new cells recruited (Cells added 1). Middle row, Cells activated following pairing (ii) and after increasing stimulus strength (iii) along with new cells recruited by the stimulus increase (Cells added II). Bottom row, images show cells activated by the alveus stimulation (orange) superimposed with those of the SC ensembles recruited by pairing-induced plasticity (Alv stim + I) and the increase in stimulus strength (Alv stim + II). Cells color-coded white belong to both the SC and alveus ensembles. B, Left, Summary showing that a larger fraction of newly added cells belong to the alveus population following LTP induction compared to those recruited by increased stimulation strength (n = 6; **, p<0.01). Right, diagram illustrating the dynamics of neuronal ensembles in this experiment. Blue and orange outlines represent the neuronal populations activated by SC and alveus stimulation, respectively. Hatched areas indicate cells that belong to both ensembles.

Given that we constrained synaptic plasticity to those SC synapses impinging onto cells depolarized by antidromic activation, the newly added cells should selectively belong to the population activated by alveus stimulation. To test this idea, we compared images of cells added to the SC ensemble to images of cells recruited by alveus stimulation (the alveus ensemble, Fig. 3A). Indeed, 83.7±5.02% of the added cells belonged to the alveus ensemble (n = 6; Fig. 3B). In contrast, increasing the size of the SC ensemble by increasing stimulus strength recruited a much smaller fraction of cells belonging to the alveus ensemble (53.1±6.23%; p<0.01; Fig. 3B). These results indicate that the addition of cells to the SC ensemble following associative plasticity does not occur randomly, but is tightly constrained by the associated alveus ensemble. Thus, these findings show that neuronal ensembles are dynamic and through associative plasticity can be enlarged to incorporate cells from a specified population.

**Figure 4:**
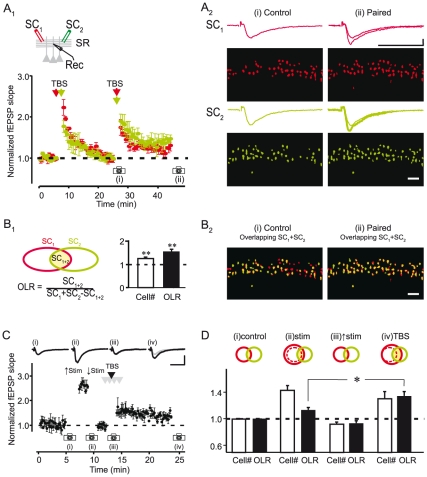
Associative LTP of two independent Schaffer collateral pathways merges the ensembles of pyramidal cells recruited by the two pathways. A_1_, Summary plot of fEPSPs showing associative LTP induced by simultaneous (paired) theta burst stimulation (TBS) of two SC pathways, while prior independent (unpaired) TBS does not cause potentiation (n = 4). Inset, recording configuration. A_2_, fEPSPs and cell ensembles evoked by each pathway (red, green) in one experiment at the times indicated on the summary plot. Scale bar, 0.2 mV and 20 ms. B, Associative LTP significantly increases the overlap ratio (OLR) of the two SC ensembles. B_1_, OLR was measured as the cells common between the two ensembles (SC_1+2_) divided by the total cells in the two ensembles (SC_1_ + SC_2_ - SC_1+2_, we subtract SC_1+2_ in order not to count cells common to both ensembles twice). Summary data plot the increase in total cells (SC_1_ +SC_2_) and OLR of the two ensembles normalized to control conditions (n = 4 slices; **, p<0.01). B_2_, Overlay of the two SC-evoked neuronal ensembles (red, green) shown in (A_2_). Yellow cells indicate neurons common to the two ensembles. (C,D) Increasing afferent input by increasing stimulus strength expands the size of cell ensembles but associative LTP causes a greater increase in overlap between two SC ensembles. C, Associative LTP was induced by pairing a weak stimulus (one TBS, black arrow) in one pathway (black traces) with a strong stimulus (four TBS, gray arrow) to the other pathway (not shown). Cell ensembles were measured under control conditions (i), following an increase in stimulus strength (ii), when stimulus strength was returned back to control (iii) and following associative LTP (iv). D, Summary data showing change in total number of cells and OLR relative to control conditions for changes in stimulus strength and associative LTP (n = 3; *, p<0.05).

We next tested whether distinct ensembles recruited by two independent SC inputs can be modified by associative synaptic plasticity to increase the fraction of cells common to both ensembles. Two SC pathways were stimulated and field EPSPs were recorded in stratum radiatum with an extracellular recording electrode (Fig. 4A_1_) while the resulting ensemble maps were visualized in the pyramidal cell layer (Fig. 4A_2_). To induce associative synaptic plasticity, we simultaneously delivered theta burst stimulation (TBS) to the two pathways. To insure that the observed potentiation resulted from the association of the two pathways, we considered only experiments in which a prior burst given to each pathway independently did not produce LTP of the field EPSP (98.7±2.1% when independently stimulated; 130.0±6.3% when simultaneously stimulated; n = 8; p<0.001; Fig. 4A_1_). Despite the independence of the two pathways as assessed with the field EPSP (see Methods), the ensembles recruited by each input were partially overlapping (Fig. 4B). We quantified the fraction of overlapping cells between two ensembles as the overlap ratio (Fig. 4B_1_): the sum of cells common to each ensemble divided by the total number of cells in both ensembles (control overlap ratio (OLR): 39.1±4.3%; n = 4). Associative LTP increased the number of cells in each ensemble (24.9±5.7%; n = 8; p<0.01; Fig. 4A_2_,B_1_) and caused a marked increase in the overlap ratio (55.8±10.3%; n = 4; p<0.01; Fig. 4B_1,2_). The large increase in overlap ratio following associative LTP resulted from the fact that a large fraction (78.5±6.2%) of the newly added cells to a given ensemble were in common with the other ensemble. Specifically, 51.2±7.3% and 27.3±7.6% of cells added were already part of the other ensemble before or appeared in the other ensemble after associative LTP, respectively.

To rule out the possibility that the increase in overlap ratio following associative synaptic plasticity can be accounted for simply by a random expansion of the neuronal ensembles we compared the changes in overlap ratio induced by associative synaptic plasticity with those induced simply by increasing the size of the ensemble via increasing the number of stimulated afferent inputs. In these experiments, we induced associative LTP in a SC pathway with TBS that was paired with a strong stimulus (four TBS) to a previously potentiated “conditioning” pathway (Fig. 4C). Increasing stimulus strength to increase the number of stimulated afferent inputs, increased the size of the field EPSP to 272.0±6.4% (n = 3; Fig. 4C), enlarged the ensemble (43.0±7.1% increase; p = 0.011; Fig. 4D) but did not significantly enhance the overlap ratio (13.7±4.2% increase; p = 0.187; Fig. 4D). Despite the fact that associative plasticity led to a smaller increase in the field EPSP (150.6±5.9%; Fig. 4C) and ensemble size (30±11.7% increase; p = 0.06; Fig. 4D), it was accompanied by a larger increase in overlap ratio (34±6.5% increase; p = 0.002; Fig. 4D; significantly larger than after increasing stimulus strength, p = 0.034). These experiments show that a simple expansion of neuronal ensembles due to increases in afferent input cannot account for the increased overlap between cell ensembles produced by associative plasticity. Thus, the expansion of an ensemble triggered by associative synaptic plasticity occurs mainly within the boundaries of the associated ensemble, thereby increasing the similarity between the two ensembles.

**Figure 5:**
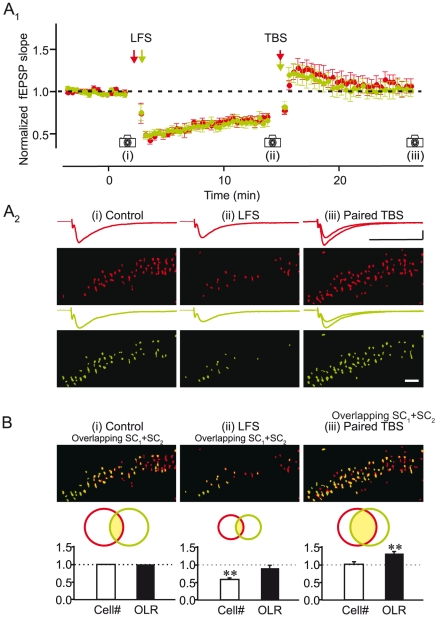
Bidirectional synaptic plasticity can merge neuronal ensembles without altering ensemble size. A_1_, Summary plot of fEPSPs showing that low frequency stimulation (LFS, 300 pulses, 1 Hz) of two SC pathways (red, green) induces LTD and subsequent paired TBS induces LTP that returns the fEPSP to control conditions. A_2_, Images and traces from one experiment collected at the time points indicated on the summary plot. LTD and LTP of fEPSPs were accompanied, respectively, by a reduction and a restoration of the size of neuronal ensembles recruited by the two SC pathways. Red and green represent the neuronal ensembles recruited by two independent SC pathways. Scale bars, 0.5 mV, 20 ms. B, Comparison of change in total number of cells and overlap between the two neuronal ensembles following LFS and subsequent paired TBS normalized to control conditions (n = 8; **, P<0.01). Schematics show the redistribution of the neuronal ensembles.

Can synaptic plasticity increase the overlap between cell ensembles recruited by two independent inputs without altering the size of the ensembles? To address this question, we took advantage of the capacity of hippocampal synapses to undergo bidirectional plasticity [12], [13]. We first induced long-term depression of two independent pathways using low frequency stimulation (1 Hz, 300 stimuli; Fig. 5A_1_). This was accompanied by a reduction of the field EPSP (74.9±4.8%; n = 16; p = 0.001; Fig. 5A_1_) and the size of the two cell ensembles (41.1±3.5% decrease; p<0.001; Fig. 5A_2_,B), while the overlap ratio was not significantly changed (8.9±7.2% decrease; n = 8; p = 0.583; Fig. 5B). Subsequent simultaneous theta burst stimulation of the two pathways increased the field EPSP (107.5±3.9%; p = 0.096; Fig. 5A_1_) and the two ensembles to approximately their original magnitudes (101.6±7.6% of control; p = 0.969; Fig. 5A_2_,B). Despite the fact that the bidirectional manipulation restored both field EPSP and cell number to control values, the similarity between the two cell ensembles was greatly enhanced (overlap ratio 30.5±8.1% increase; p = 0.007; Fig.5B). These results thus show that although bidirectional plasticity can lead to no net change in overall synaptic strength, it generates a lasting transformation of cell ensembles representing the two inputs. This transformation reflects a redistribution of cells recruited by each input that ultimately increases the similarity in the representation of the two inputs.

## Discussion

We have taken an approach to understand how the output of populations of cells is shaped by experience. We used imaging to visualize the activity of individual neurons within a population in order to understand how associative plasticity might reassign cells between different ensembles. We show that two different sets of afferents are represented by unique ensembles of cortical neurons, which become more similar following associative plasticity of the two inputs. Furthermore, because of the capacity of synapses to undergo bidirectional plasticity we show that the enduring trace is not necessarily reflected by a net change in synaptic strength, but rather by a redistribution of the neurons representing a given input.

Taken together our data show that the representation of afferent inputs by active neuronal populations is fluid and dynamic and shaped by associative synaptic plasticity.

Activity-dependent synaptic plasticity is widely viewed as a physiological mechanism that plays a fundamental role in learning and memory. Indeed, the basic properties of LTP such as its persistence, input specificity and requirement for the association of pre- and postsynaptic activity are consistent with associative forms of learning and memory [14]. While changes in afferent synaptic strength are considered the initial mechanism by which “memory traces” are encoded and stored [15], modifications in synaptic strength are ultimately represented by the firing activity (output) of postsynaptic neurons. ln this study we examined how experience-dependent changes in synaptic strength can alter the ensemble of postsynaptic neurons brought to spike threshold within a cortical circuit. We found that associative synaptic plasticity resulting from the co-activation of distinct afferent inputs leads to the generation of novel ensembles whose composition is a combination of the two associated ensembles.

In the simplest case, our results show that the unique but partially overlapping ensembles of CA1 pyramidal cells activated by different afferent fibers become more similar to one another following associative LTP. The convergence of SC fibers onto pyramidal cell dendrites and the selective strengthening of converging synaptic inputs induced by associative LTP best explain this remapping of neural ensembles. NMDAR-dependent LTP induced by the pairing of weak tetanic stimulation delivered to two afferent pathways preferentially enhances the strength of synaptic inputs that converge onto common cells [7], [14]. This reflects the cooperative action of combining synaptic inputs to achieve sufficient postsynaptic depolarization for the induction of NMDAR-dependent LTP. Thus, the selective strengthening of two unique afferent pathways that converge onto the same postsynaptic cell increases the likelihood that both pathways will be strong enough to bring the cell to spike threshold. In terms of active cell ensembles, cells recruited to fire in response to one afferent pathway will be more likely to be recruited by an initially subthreshold pathway following associative LTP. The overall consequence is an increase in the similarity of cell ensembles recruited by the distinct, but converging, afferent inputs.

A popular model for associative learning and memory relies on the notion that cell assemblies that are repeatedly active at the same time will become “associated” such that activity in one set of cells facilitates the activity of another [16]. Our results describing the linking of different afferent inputs to a more similar output of neuronal ensembles offers a compelling example of how associative synaptic plasticity modifies cell assemblies in the hippocampus.

## Methods

### Slice preparation and electrophysiology

Hippocampal slices (400 µm) were prepared from Sprague Dawley rats (P14–28) in accordance with UCSD IACUC guidelines (Approval ID S99077R). Transverse slices were cut in ice-cold cutting solution containing (in mM) 83 NaCl, 2.5 KCl, 0.5 CaCl_2_, 3.3 MgSO_4_, 1 NaH_2_PO_4_, 26.2 NaHCO_3_, 22 glucose, and 72 sucrose, and incubated in an interface chamber at 34°C for 30 min and at room temperature thereafter. Recordings were performed at 30–32°C in artificial cerebrospinal fluid (aCSF) containing (in mM): 119 NaCl, 2.5 KCl, 1.3 NaHPO_4_, 1.3 MgCl_2_, 2.5 CaCl_2_, 26 NaHCO_3_, 11 glucose (equilibrated with 95% O_2_ and 5% CO_2_). Experiments using pairing of alveus and SC stimulation were done in a modified aCSF containing (in mM): 119 NaCl, 5 KCl, 4 CaCl_2_, 4 MgSO_4_, 1 NaH_2_PO_4_, 26.2 NaHCO_3_, 22 glucose, and 0.1 picrotoxin, equilibrated with 95% O_2_/5% CO_2_. The high divalent concentrations (4 mM Ca^2+^ and 4 mM Mg^2+^) were used to suppress epileptiform activity in the presence of the GABA_A_ receptor antagonist picrotoxin. One radial cut was made to separate the CA3 and CA1 regions.

Stimulation (0.2 ms) was applied using bipolar electrodes. Glass pipettes filled with ACSF (1–2 MΩ) were used to record field EPSPs. Stimulating electrodes were placed either in the alveus to antidromically stimulate pyramidal neurons, or in stratum radiatum to activate SC inputs. fEPSPs were recorded with a MultiClamp700A amplifier (Axon Instruments/Molecular Devices, Foster City, CA), filtered at 2kHz and digitized at 10kHz. Data acquisition and analysis were performed with Axograph 4.9 (Axograph) and IGOR Pro 4 (Wavemetrics, Lake Oswego, OR) software. Theta burst stimulation consisted of 5 SC stimuli delivered at 100 Hz. Independence of two SC pathways was evaluated by comparing the digital sum of the initial slopes of the fEPSPs of the two pathways with those evoked by stimulating both pathways simultaneously. Stimulus intensity of SC pathways was adjusted to evoke responses that were 40–50% of the peak amplitude of the maximum fEPSP for LTP experiments, and 70–80% of maximum for LTD experiments. The alveus layer was stimulated at an intensity that maximally activated neurons in the pyramidal cell layer. Data are presented as mean±SEM. Student's *t*-test and one-way ANOVA were used to determine statistical significance.

### Imaging

For population calcium imaging, Oregon Green BAPTA-1 AM (∼400 µM, with 1% Pluronic F-127. Molecular Probes, Eugene, OR) was pressure injected into the pyramidal cell layer. Slices were allowed to recover for 20 min before imaging experiments commenced. Recordings of calcium transients were combined with simultaneous fEPSP recordings (20x objective), or loose-seal patch clamp recordings from single cells (60x objective). Image acquisition (494 nm excitation, 2×2 binning, 15–20 Hz) was carried out with a cooled-CCD camera system (T.I.L.L. Photonics). Image processing and analysis were performed with ImageJ (National Institutes of Health, Bethesda, MD). Activity maps of cell ensembles were constructed from 4–6 stimulus trials. All images from an individual experiment were processed identically. The first 3–4 image frames (∼200 ms) following the stimulus were averaged to generate the peak dF/F signal for individual trials (without background subtraction). Individual peak dF/F images were than averaged together to represent the cell ensembles activated by each input pathway. The resulting peak dF/F images were bandpass filtered (to reduce diffuse signals) and smoothed. Cells were detected from these processed images using a template-based detection criterion based on their size (∼20 µm diameter) and intensity (>50% of background) to yield a final binary image of active cells.
